# Selection on plasticity of seasonal life-history traits using random regression mixed model analysis

**DOI:** 10.1002/ece3.60

**Published:** 2012-04

**Authors:** Jon E Brommer, Pekka Kontiainen, Hannu Pietiäinen

**Affiliations:** Bird Ecology Unit, Department of Biosciences, University of HelsinkiP.O. Box 65 (Viikinkaari 1), FIN—00014, Finland

**Keywords:** Bird, clutch size, natural selection, phenotypic plasticity, reaction norm

## Abstract

Theory considers the covariation of seasonal life-history traits as an optimal reaction norm, implying that deviating from this reaction norm reduces fitness. However, the estimation of reaction-norm properties (i.e., elevation, linear slope, and higher order slope terms) and the selection on these is statistically challenging. We here advocate the use of random regression mixed models to estimate reaction-norm properties and the use of bivariate random regression to estimate selection on these properties within a single model. We illustrate the approach by random regression mixed models on 1115 observations of clutch sizes and laying dates of 361 female Ural owl *Strix uralensis* collected over 31 years to show that (1) there is variation across individuals in the slope of their clutch size–laying date relationship, and that (2) there is selection on the slope of the reaction norm between these two traits. Hence, natural selection potentially drives the negative covariance in clutch size and laying date in this species. The random-regression approach is hampered by inability to estimate nonlinear selection, but avoids a number of disadvantages (stats-on-stats, connecting reaction-norm properties to fitness). The approach is of value in describing and studying selection on behavioral reaction norms (behavioral syndromes) or life-history reaction norms. The approach can also be extended to consider the genetic underpinning of reaction-norm properties.

## Introduction

**Figure 1:**
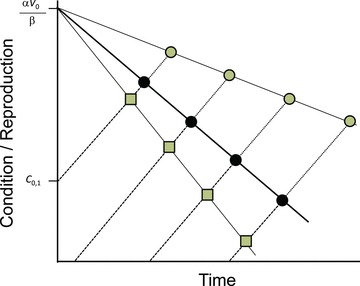
Illustration of the main theoretical background of the reaction-norm concept in terms of two seasonal life-history traits. As the season advances (Time), environmental conditions and thus the potential clutch size *C* increase (Equation [1]; dotted line), but individuals experience different trajectories characterized by the initial condition *C*_0_. Because the reproductive value of offspring *V* declines seasonally (Equation [2]), there is an optimal switching curve (solid line) that describes when an individual should stop increasing in condition and reproduce (filled dots). This switching curve is the optimal reaction norm maximizing *V*(*t*)*C*(*t*), showing a seasonal decrease in reproduction (Equation [3]). Timing reproduction earlier has fitness costs, because reproductive output decreases (gray squares), more so than offspring reproduction increases. Delaying reproduction increases reproductive output (gray dots), but has fitness costs because late-produced offspring are of lower value.

Reaction-norm theory states that covariance of life-history traits occurs when individuals experience a heterogeneous environment, where the optimal combination of life-history traits depends on the environmental context an individual finds itself in (Stearns 1992; Kozłowski 1992). For example, in practically all temperate avian species, birds that lay later in the breeding season have a lower clutch size than individuals which produce earlier in the breeding season (Klomp 1970). The negative covariance of these two life-history traits (clutch size and laying date) is generally viewed as a reaction norm in response to environmental heterogeneity. Under poor environmental conditions, it is optimal for an individual's fitness to reproduce late in the season and produce a small number of eggs compared to individuals that experience good conditions (Fig. 1; Daan et al. 1990; Rowe et al. 1994; Brommer et al. 2002a). Consider, for example, a linear increase over time *t* in the potential clutch size *C* of individual *i* as 

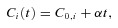
1
where potential clutch size *C* can be considered a common currency to express differences across individuals in the environmental conditions they experience, for example, due to the food supply in their territory. All interindividual variation in this example is fully due to variation in the initial potential clutch size experienced by individual *i*, C_0,*i*_ (but see Rowe et al. 1994 and Brommer et al. 2002a for other scenarios). The reproductive value *V* of an egg produced at time *t* declines as 

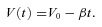
2

The optimal clutch size C* and laying date t* are then found by maximizing the product *C*(*t*)*V*(*t*) for every individual *i*, which generalizes (details in Brommer et al. 2002a) to 

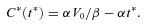
3

Hence, the individually optimized reaction norm is a decline in clutch size with advancing laying date at rate α. Although this concept is here illustrated with clutch size and laying date, the same arguments apply to other seasonal life-history traits such as, for example, size at maturation (related to fecundity) and seasonal developmental time in insects (Kawecki and Stearns 1993).

The above example of the theory behind reaction norms implies that individuals that do not follow the optimal reaction norm suffer reduced fitness compared to those that follow the norm. In the above example, if an individual advances laying date relative to the reaction norm (gray squares in Fig. 1), fecundity is reduced and this reduction exceeds the gain in reproductive value of the offspring produced. If the individual delays and produces a larger clutch size relative to the reaction norm (gray dots in Fig. 1), fitness costs occur in terms of reduced reproductive value of the offspring produced which outweigh the increase in fecundity. Thus, evaluation of empirical reaction-norm data should reveal selection on the properties describing the reaction norm. In particular, theory predicts that the slope of the trait–trait relationship should be under selection. Despite the intuitive appeal of the theory, empirical demonstration that a certain covariation of traits is under selection is challenging. This is because it requires two steps:

1. Quantification of the variation across individuals or genotypes in their reaction-norm properties. In its simplest form, a reaction norm is a linear function (Trait Y = elevation + slope × Trait X), thereby requiring estimation of the variance of at least two properties. Whereas the “elevation” is clearly related to the mean value of the trait, the “slope” (within-individual covariance between two traits) is an emergent property of the observed data and is therefore sensitive to choice of statistical procedure used to estimate it. Some of these procedures will have more power to describe variation in slope than others.
2. Selection on a reaction norm requires the calculation of the fitness of these reaction norm properties, rather than the fitness of the expression of a trait itself (for more discussion on this notion, see Weis and Gorman (1990), and Spitze and Sadler (1996)). Because fitness is a trait of the individual, this implies that, in statistical terms, a description of reaction-norm fitness must allow for the covariance between fitness and reaction norm slope, where the latter is an emergent property (a “meta-trait”) defined by the within-individual covariance of two traits.

Brommer et al. (2003) noted that for labile traits for which expression changes across an individual's lifetime, measurement of life-history traits (under varying environmental conditions) and lifetime fitness can be obtained for each individual. Hence, selection on plasticity can be calculated for such traits. Nevertheless, calculation of selection on plasticity has thus far been based on regression of estimates of an individual's reaction-norm slope (describing the covariance between two traits or between a trait and an environmental value) on an individual's fitness, thereby doing stats-on-stats, either on slope estimates derived from linear regression (Brommer et al. 2003; Nussey et al. 2005a) or derived from a linear mixed model (as Best Linear Unbiased Predictor [BLUP] values, Brommer et al. 2005; Nussey et al. 2005b). Linear regression of data on each individual requires a certain minimal number of repeated records per individual (e.g., five in Brommer et al. 2005; four in Nussey et al. 2005a), thereby discarding a potentially sizeable proportion of the data consisting of observations on individuals with relatively few repeated records. In terms of selection analysis, this introduces a possible bias as only the longest lived individuals are included. Mixed model BLUP values of a response variable are based on the assumption that all covariates that may have an effect on the response variable have been included (Pinheiro and Bates 2000). Hence, using BLUP values to explore covariation with another trait (e.g., fitness), violates the assumption under which they were generated in the first place. Typically, both approaches ignore the often large uncertainty inherent in estimating slope when quantifying selection on the slope estimate, which may produce spurious estimates of selection.

Here, we use random regression mixed models to calculate both the reaction-norm properties and selection on these in an integrative manner that circumvents the above-described problems. Random regression is an implementation of the infinite-dimensional model (e.g., Kirkpatrick and Heckman 1989), which presents a simplification of the character state approach by allowing trait variation to be described as a function of a covariate. As all mixed models, random regression is robust for analyzing unbalanced data, which data from natural populations often are (Crawley 2002). Here, we argue that a mixed model random regression can be used to explore variation in the properties underlying a trait–trait reaction norm. Random regression mixed models have mostly been used in the study of trait–environment relationships in natural populations, both on the phenotypic and genetic levels (reviewed by Nussey et al. 2007), but have—to our knowledge—not been implemented in the study of covariance between traits. Furthermore, we demonstrate how multivariate random regression can be used to estimate selection on reaction-norm properties. Multivariate random regression models have been rarely used in ecology, with the exception of exploration on how the covariance between traits changes over an environmental gradient (e.g., Robinson et al. 2008) and in formal comparison of trait–environment reaction norms in two populations (Husby et al. 2010).

We illustrate our approach by a consideration of selection on the properties describing the clutch size–laying date relationship. Our study species, the Ural owl (*Strix uralensis* Pall.), experiences a highly variable environment, and shows high plasticity in clutch size (one to eight eggs) and seasonal timing of laying (more than 2 months) (e.g., Pietiäinen 1989). Previous work (based on linear regression and analysis of covariance) has shown that there is variation across Ural owl females in their clutch size–laying date relationship in terms of elevation and slope (Brommer et al. 2003). Here, we quantify the variation in these properties and selection on them using random-regression analysis. We discuss the applicability of this method in the study of phenotypic integration of behavioral and life-history traits.

## Methods: Random-Regression Approach

We outline the model approach in terms of the analysis of clutch size and laying date. We here adhere to this specific scenario, instead of a more generic one, in order to facilitate understanding the approach and linking it to the example scenario presented in this paper and the code for implementing this model, which is presented in the Supporting Information. Nevertheless, the approach is applicable to also other combinations of traits that are expressed repeatedly during an individual's lifetime.

Model construction and evaluation was performed in two stages. We started by modeling the clutch size–laying date relationships, such that 

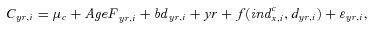
4
where *c_yr,i_* denotes the clutch size of female *i* in year *yr*. The “µ_c_” fits the overall fixed effects mean clutch size, *Age*F*_yr,i_* is a fixed effect that denotes the factor age of individual *i* in year *yr*, *d_yr,i_* the laying date of female *i* in year *yr*, and *b* the fixed-effect slope of clutch size as a function of laying date. Any annual variation that is not explained by the fixed effects is modeled by the random effect *yr*. The random regression element is given by the polynomial function 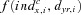 in Equation (4), which specifies the individual-specific deviations from the fixed-effect slope *b* of clutch size on laying date. For each individual *i*, a polynomial function of increasing order *x* is specified. Thus, when *x*= 0, the variance across individuals (*ind*_0_) is estimated at the average laying date value. When *x*= 1, the variance in the coefficients *ind*_0_ (elevation) and the variance in *ind*_1_ (slope) of the function *ind*_0_+*ind*_1_*d_year_* is estimated and the covariance between these, and so on for higher order polynomials. Equation (4) is a standard random regression model, with the exception that typically an environmental variable is used as explanatory variable (e.g., Schaeffer 2004; Nussey et al. 2007).

To decide the order *x* of the random regression, we assumed that the most parsimonious random regression model was reached when higher order polynomials did not achieve a significant increase in log-likelihood. That is, the order *x* of the random regression polynomial function was increased stepwise, and its significance was tested by a likelihood ratio test, which is two times the difference in log-likelihood between hierarchically nested models, tested against a chi-square distribution assuming that the degrees of freedom are given by the additional number of (co)variances estimated. Based on the most parsimonious random regression model, estimates of variance in clutch size at each laying date and its approximate standard error can be calculated following Fischer et al. (2004).

### Selection on reaction-norm properties

Having determined the most parsimonious order of the polynomial random regression, we extended the univariate random regression (Equation[4]) to a bivariate one by in addition to Equation (4) considering that 

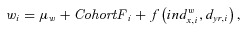
5
where *w_i_* is the relative fitness of individual *i*. The µ*_w_* denotes the overall fixed effect mean fitness, and *Cohort*F*_i_* a fixed effect denoting the year of first breeding of individual *i*, which is entered to correct for temporal variation in fitness and the possible truncation of lifetime fitness in the last cohorts considered in the analysis. The bivariate element in the random regression is specified by the polynomial 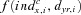. Thus, for order *x*, the variances in 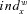 and 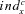 are both estimated, in addition to all possible covariances between these parameters. Nevertheless, because each individual has only one estimate of lifetime fitness, only the variance in elevation (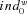) is estimable and all other variances (including the residual variance) must be constrained to zero. Thus, for a first-order random regression, the mixed model procedure needs to estimate the (co)variances (written in matrix form, omitting the upper diagonal that is symmetric to the lower diagonal). 

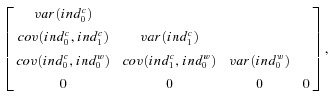
6
where 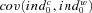 denotes the selection differential on the elevation of the clutch size–laying date relationship (*S*_elevation_) and 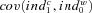 the selection differential on the slope of the clutch size–laying date relationship (*S*_slope_). These selection differentials can be expressed as a selection gradient by dividing them with 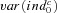 and 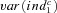, respectively, or as selection intensity (standardized selection gradient) by dividing them with the square root of these variances.

Higher order polynomials can be introduced as an extension of this matrix. Thus, bivariate random regression allows estimating directional selection on the properties of a polynomial individual-specific relationship between labile life-history traits. Testing the formal significance of the selection differential can be performed by constraining the appropriate covariance to zero and performing a likelihood ratio test between the unconstrained and constrained models. The procedure does not, however, allow description of nonlinear selection. In order to evaluate the potential for nonlinear selection on these properties, we suggest investigation of the map of relative fitness on the BLUP values for all reaction-norm properties. Under the paradigm of optimal reaction norms, one would expect that variation in slope may be under stabilizing selection, which can hereby be graphically evaluated.

All random regression models were solved using Restricted Maximum Likelihood (REML) in the programe ASReml (VSN International). This programe uses the delta method (see e.g., Lynch and Walsh 1998) to estimate the standard errors of functions of variances. The code used in this paper and example file of data is provided in the Supporting Information. In the implementation of the above equations in AsReml, the following need to be observed. (1) In random regression with a linear covariate, the variances of first-order and higher order terms are dependent on the scaling of the covariate (e.g., Pinheiro and Bates 2000; Schaeffer 2004), which is dependent on the software used to implement the models. AsReml standardizes the covariate such that its minimal value is –1 and the average of the covariate values is zero. Hence, variances in elevation are defined for the average of all the covariate values (which may or may not equal the average covariate when taking the number of observations into account). Random-regression slope is then defined as change in response variable per unit equal to the difference between minimum and mean value of the covariate. (2) The within-individual variance and variance in slope(s) of lifetime fitness cannot be constrained to exactly zero and must, in practice, be constrained to a very small value. See the Supporting Information for additional details.

## Example of the Random-Regression Approach: Ural Owl Clutch Size–Laying Date Reaction Norms

### Ural owl data

A nest-box breeding population of Ural owls was studied in Päijät-Häme, southern Finland, in an area of 1500 km^2^ during 1977–2009. Laying date of the first egg was established either by finding the incomplete clutch and calculating back assuming that eggs are laid at 2 days interval or by backdating first the hatching date of the nestlings (based on a daily growth curve) and assuming an incubation period of 32 days for the first egg (largest nestling). Any error in establishing laying date using these approaches is likely to be small in comparison to the length of the period of laying, which spans 2 months. See Pietiäinen (1989) and Kontiainen et al. (2010) for details. Each year, practically all Ural owls females were caught, either during incubation or when caring for their offspring. All females were aged by plumage characteristics to age categories of 1, 2, or ≥3 years old (Pietiäinen and Kolunen 1986) and were ringed with a metal ring in order to allow lifelong identification. All offspring were ringed and all boxes were checked after fledging to verify successful fledging of offspring.

### Data used in the analysis

For the analyses, individuals that started their breeding career during the study period were included. We thus omitted individuals trapped for the first time in 1977, but assumed that all females caught for the first time in one of our boxes from 1978 onwards were first breeders. We included data on clutch size and laying date on all females that started their breeding career between 1978 and 2008 (31 years). We included age as a fixed-effect factor with three levels (1, 2, 3+ years old). Laying date was standardized relative to the all-time median laying date of 31 March (= 0), such that 1 April was 1 and 30 March was –1.

As an estimate of lifetime fitness, we used the total number of fledglings that a female has produced during her entire breeding career, which in this population correlates with the number of descendants after several generations (Brommer et al. 2004). Relative fitness *w_i_* of Equation (5) was calculated by dividing an individual's lifetime production of fledglings by the overall mean lifetime production of fledglings (fitness is relative to an average of unity).

We could analyze the clutch size–laying date relationships and their fitness consequences of 361 Ural owl females with 1115 breeding attempts (3.1 records of clutch size and laying date per female on average). All females with known laying date and clutch size that had bred at least once in our study population were included. Results did not change qualitatively when animals with only one breeding record were excluded.

## Results: Interindividual Variation in the Clutch Size–Laying Date Relationship

**Figure 2:**
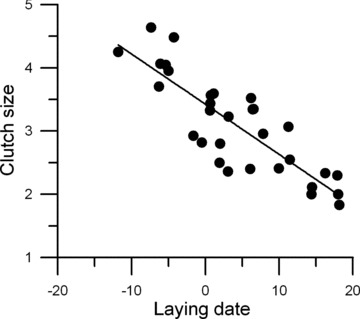
Yearly mean in clutch size and laying date for all Ural owl females included in the analysis. Laying date was expressed in days relative to its long-term median (31 March). Data consist of 1114 observations collected over 31 years. Line displays the linear regression (coefficients and test reported in the text).

The annual mean Ural owl clutch size decreased approximately linearly with advancing laying date (Fig. 2; Linear regression, intercept: 3.39 ± 0.098, *t*_30_= 34.5, *P* < 0.001; slope: –0.087 ± 0.014, *t*_30_=–6.3, *P* < 0.001; slope^2^: 0.00086 ± 0.0011, *t*_30_= 0.78, *P*= 0.44). Random regression analysis (Table 1) showed that there was substantial variation in clutch size across years that was not explained by variation in laying date or by age (model 2). Further, there was clear evidence of variance across females in all the elevation *ind*_0_ terms (model 3), linear slope terms *ind*_1_ (model 4), and quadratic slope terms *ind*_2_ (model 5) of their clutch size–laying date relationships. The model that allowed individual-specific cubed (*ind*_3_ term) clutch size–laying date relationships (model 6, Table 1) was not a further improvement in model fit. We thus assumed that a second-order polynomial described the variation across individuals in their clutch size–laying date relationship most parsimoniously. Investigation of the fixed effects estimated under the final model (Table 1) showed that clutch size declined with 0.06 egg per day of advancing laying date and that 1-year-old females tended to produce a smaller clutch size (although not significant, this effect was kept in the model).

**Table 1:** Hierarchical random regression linear mixed models of clutch size as a function of laying date of increasing polynomial order and their significance, as specified by Equation (1). Reported in the upper part of the table are the REML variances (covariances are reported in the text). The significance of higher order random regression is tested by a likelihood ratio test comparing the likelihood of each model to the hierarchical lower one. Residual variance is denoted for each model, and the coefficients of the random regression (Equation [1]) are listed in increasing order such that *ind*_1_ is the first-order (linear) coefficient, *ind*_2_ the second order. For each test, the degrees of freedom (df) are given by the number of additional variances and covariances that are estimated. The most parsimonious model is presented in bold. The lower part of the table presents the fixed effects and their standard error of the most parsimonious model, with their significance tested using *F*-tests.

		Random regression variances					Test between models			
Model	Residuals	Year	*ind*_0_	*ind*_1_	*ind*_2_	*ind*_3_	LogL	χ^2^	df	*P*
1	0.735	–	–	–	–	–	–397.2			
2	0.575	0.179	–	–	–	–	–295.2	204.0	1	<0.001
3	0.452	0.165	0.126	–	–	–	–264.8	60.8	1	<0.001
4	0.413	0.161	0.122	0.417	–	–	–256.7	16.2	2	0.003
5	**0.397**	**0.156**	**0.141**	**0.240**	**0.975**	**–**	**–251.7**	**10.0**	**3**	**0.019**
6	0.391	0.156	0.146	0.234	1.083	0.487	–249.3	4.8	4	0.31
Fixed effect	Estimate		df (nom)	df(den)	*F*	*P*				
μ	3.38 ± 0.080									
Laying date	–0.062 ± 0.0032		1	382.3	377.3	<.001				
Age	1: –0.22 ± 0.14									
	2: 0.032 ± 0.10		2	983.6	1.3	0.285				

**Figure 3:**
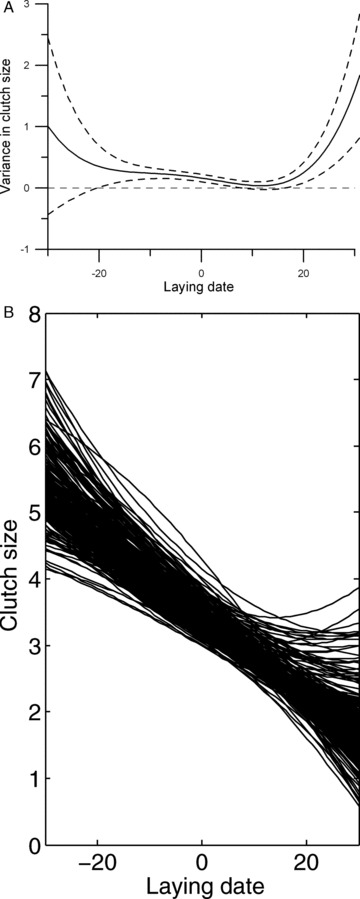
(A) Plot of the variance in clutch size (solid line) and its approximate 95% confidence interval (dashed line) as a function of laying date. Values based on statistics of model 5 in Table 1 and applying the results of Fisher et al. (2004). (B) Plot of the reaction norms based on the fixed effects of elevation and slope and the Best Linear Unbiased Predictor (BLUP) values of model 5 for the individual females’ reaction norms (Table 1).

To visualize the thus-modeled relationship between clutch size and laying date, we plotted the variance in clutch size as a function of laying date (Fig. 3A) based on the derivations derived by Fischer et al. (2004). In addition, we plotted the individual clutch size–laying date relationships for the females, based on the BLUP values from model 5 (Table 1) of *ind*_0_, *ind*_1_, and *ind*_2_ (Fig. 3B). Both methods showed clearly that there was considerable crossing of the reaction norms such that variance in clutch size across individuals was minimal around the average laying date (0) and high for both early and late clutches.

### Selection on the clutch size–laying date relationship

Selection could be detected on all parameters that described the clutch size–laying date relationship (Table 2). Not surprisingly, the selection differential on the elevation of clutch size (expected clutch size at the average of laying date values) showed clear selection for increased clutch size. There was an insignificant selection differential for a steeper (more negative) slope of the clutch size–laying date relationship. Lastly, there was significant positive selection for the second-order random regression term of the clutch size–laying date relationship. That is, female whose clutch size–laying date relationship was more concave had higher lifetime fitness. Because these selection differentials take into account the whole (co)variance matrix (Table 2), selection on the second-order term occurred in addition to the selection on its correlate elevation. Because the bivariate random regression can only describe linear selection, we investigated the plots of relative fitness versus the BLUP values for the reaction-norm properties to find evidence for stabilizing selection on linear slope or elevation. One may expect stabilizing selection on the reaction norm in case both shallower and steeper slopes reduce fitness (e.g., Fig. 1). Nevertheless, these plots revealed no evidence for stabilizing selection (Fig. S1).

**Table 2:** Variance–covariance matrix (with SE) of the parameters involved in bivariate random regression of clutch size and lifetime fledgling production (LFP) as a function of laying date. LFP was expressed as relative fitness, by dividing it with its phenotypic mean (mean fitness of 1). The diagonal presents the variances in elevation (*ind*_0_), linear slope (*ind*_1_), and quadratic slope (*ind*_2_), and LFP. Covariances are below the diagonal. The last row therefore presents the selection differential (*S*) on the reaction-norm properties. Correlations are reported above the diagonal. Model log-likelihood is –342.19. Significance of selection was tested using a likelihood ratio test between this full model and the model where the respective element of the matrix was constrained to zero. The log-likelihood (LogL) of the constrained model and the test statistics is reported in the bottom part of the table, together with the intensity if selection (i.e., *S* divided by the standard deviation of the respective parameter). The denominator degrees of freedom cannot be estimated numerically for this model and were conservatively equaled to the number of individuals.

	Bivariate random regression (co)variances				
	*ind*_0_	*ind*_1_	*ind*_2_	LFP	
*ind*_0_	0.129 ± 0.041	–0.407 ± 0.23	0.346 ± 0.23	0.394 ± 0.11	
*ind*_1_	–0.074 ± 0.043	0.260 ± 0.15	0.218 ± 0.33	–0.194 ± 0.16	
*ind*_2_	0.099 ± 0.095	0.089 ± 0.13	0.645 ± 0.33	0.516 ± 0.18	
LFP	0.104 ± 0.031	–0.073 ± 0.056	0.304 ± 0.092	0.539 ± 0.042	
Fixed effect on clutch size		df (nom)	df (den)	*F*	*P*
μ	3.35 ± 0.079				
Laying date	–0.060 ± 0.0031	1	361	379.4	<0.001
Age	1: –0.19 ± 0.14				
	2: 0.027 ± 0.10	2	361	0.88	0.42
Fixed effect on LFP		df (nom)	df (den)	*F*	*P*
μ	1.54 ± 0.19				
Cohort year		26	361	1.40	0.095
Parameter	Intensity of selection (*i*)	LRT			
		LogL	χ^2^	df	*P*
*ind*_0_	0.29	–340.6	15.9	1	<0.001
*ind*_1_	–0.14	–335.0	1.83	1	0.17
*ind*_2_	0.38	–340.0	13.4	1	<0.001

### Interpretation of the findings

We find that female Ural owls differ in the elevation, linear and quadratic slopes of their clutch size–laying date relationships. This variation across individuals is important, because it creates the potential for selection to shape the plasticity in clutch size as a function of laying date. We indeed show that selection operates on this plasticity, such that (1) females enjoy greater fitness when they have a larger elevation. That is, a larger clutch size at the mean value of observed laying dates, which is a close correlate of an individual's mean clutch size (cf. Brommer et al. 2003), (2) females whose clutch size declines steeper with advancing laying date and who show a stronger nonlinear (concave) curvature in their clutch size–laying date relationship have higher fitness. Inspection of the interindividual clutch size–laying date relationship estimated by random regression (Fig. 3) shows that individuals with a concave curvature are those which have a relatively moderate clutch size at the early laying dates (before the median) and a relatively low clutch size during late laying dates (after the median). In the Ural owl, fledging success in brood sizes exceeding five offspring generally is poor, because hatching asynchrony increases rapidly with increasing clutch size (Kontiainen et al. 2010). For example, in a brood of 5, the smallest offspring is already 4–6 days younger than its oldest sibling and mortality of the youngest nestling in such a large brood is likely (Kontiainen et al. 2010). Therefore, the production of large clutch sizes adds little to lifetime fledgling production. In addition, late broods are typically found under poor environmental conditions (Pietiäinen 1989) and it may be a good strategy to reproduce conservatively under those conditions. Thus, we believe that our selection analysis captures biologically plausible processes in this species. The main finding is, nevertheless, that there is selection on properties that describe the plasticity of seasonal adjustment in clutch size. Provided part of this latter variation across females in plasticity is heritable, selection has the potential to shape the clutch size–laying date relationship in this species.

## Discussion

Theory states that the covariation in seasonal life-history traits such as clutch size and laying date arises from individual-specific fitness optima, suggesting that deviation from the reaction norm reduces fitness. Here, we advocate an approach based on random regression to first describe and test whether there is variation across individuals in the properties describing a trait–trait reaction norm and to further test for selection on these properties. We show that this approach indeed is effective in describing variation in Ural owl clutch size–laying date relationships and detects selection on the reaction-norm properties. Using a random regression mixed model to describe trait–trait reaction norms and selection on them avoids having to first extract information on reaction-norm properties from individual-specific regression and then regressing these estimates on fitness (e.g., Brommer et al. 2003; Nussey et al. 2005a). Hence, uncertainties in the estimate of an individual's reaction-norm properties are incorporated and selection on the mean trait (i.e., elevation) is considered simultaneously to the within-individual covariation between traits as well as their codependency by considering the complete matrix of variances and covariances between the trait under investigation and fitness. The approach works especially well on data consisting of repeated measures of traits on a set of individuals, of which the seasonal life-history traits in the case scenario considered is but one example.

Selection acts on phenotypes, and, in case of phenotypic plasticity, the phenotype is an environment-specific realization of the genotype. Empirically measuring the fitness of the reaction norm requires integrating the fitness of all the phenotypes produced by a genotype over the entire environmental gradient (Weis and Gorman 1990). However, in the case of labile traits, we consider trait expression by a set of individuals. Although each individual is a unique genotype, the variance between individuals in the properties describing the trait–trait reaction norms includes both the additive genetic variance and also other, nonheritable, consistent differences across individuals (so-called permanent environmental effects, Lynch and Walsh 1998). In our example, environmental effect associated with individuals may arise because Ural owls are strongly territorial and essentially breed their entire career in the same territory (Saurola 1987). Thus, variation across territories is confounded with variation across individuals and it is therefore possible that the variation in reaction-norm properties we here document is entirely caused by environmental differences across individuals. Nevertheless, provided sufficient pedigree information is available, a random regression animal model can be used to further partition the phenotypic properties into their additive genetic versus permanent environmental components (Nussey et al. 2007). Such a random regression animal model would allow estimation of the additive genetic (co)variance matrix G for the reaction-norm properties.

### Nonlinear selection on reaction norm properties

A major downside of the bivariate random regression approach in quantifying selection on plasticity is that it does not allow for nonlinear selection. For a single trait, nonlinear selection is estimated by calculating the covariance between the square of the trait and relative fitness (Falconer and MacKay 1996). The reaction-norm slope is an emergent property determined by the within-individual covariance between the traits and cannot be directly measured. Selection on higher order polynomial terms, as we found in our example case for the second-order polynomial of the Ural owl clutch size–laying date relationship, refer to linear selection on specific curvature of the reaction norm itself rather than the shape of natural selection. Stabilizing selection on slope would be expected in case the reaction norm is maintained by selection. Nevertheless, there is generally a component of directional selection, except in the very restrictive case of completely symmetrical reductions in fitness if the slope deviates from the optimal reaction norm. Furthermore, comparative studies underline that directional selection is typically stronger than stabilizing selection (Kingsolver et al. 2001; but see Stinchcombe et al. 2008). Investigation of the mapping of fitness to the BLUPs of the reaction-norm properties, as we did here, should reveal whether stabilizing selection on reaction norms may occur (although it does not allow a formal test). We advice that the estimates of selection on reaction-norm properties are considered with caution, but also emphasize that alternative approaches may lead to spurious findings, especially when the uncertainty of the slope estimate is ignored.

### Applications of the random-regression approach in evolutionary ecology

The approach used here to quantify selection on trait–trait reaction norms is equally useful for reaction norms of trait versus environment. The typical use of random regression mixed models is to model the covariance of a trait and an abiotic variable. For example, reaction norms in laying date and temperature have been explored in several studies in wild populations (e.g., Brommer et al. 2005, 2008; Nussey et al. 2005b; Charmantier et al. 2008; Husby et al. 2010). Such random regression models can be readily extended to a bivariate form where fitness is also considered (Equation [6]) in order to analyze selection on the trait–environment reaction-norm properties. Understanding selection on trait–environment plasticity may be crucial, because under environmental change organisms may be selected to alter the plasticity in their trait–environment relationship, rather than merely their mean trait value. For example, Nussey et al. (2005b) showed that selection on the BLUPs of the slope of laying date–temperature relationships of great tits *Parus major* in the Netherlands increased as the local spring climate warmed during the last decades.

Studies that consider repeated measures of different behaviors collected on a set of individuals may benefit from the approach outlines in this paper. The level of integration of several behaviors within an individual is increasingly receiving attention in ecology and evolutionary biology, because it captures “‘behavioural syndromes,” consistency in behaviors across different contexts (Sih et al. 2004). Under this paradigm, correlated behaviors (e.g., between “boldness” and “aggression”) arise because individuals are relatively inflexible with respect to environmental conditions (Réale et al. 2007). Hence, explicit consideration of “behavioural reaction norms” and understanding selection on their properties has been recently advocated as a potentially fruitful line of research for understanding how natural selection shapes behavioral syndromes (Dingemanse et al. 2010). In particular, the notion that behavioral syndromes are maintained in the population because plasticity, that is, reaction-norm slope, is genetically constrained (Sih et al. 2004) as opposed to being shaped by selection can be explicitly explored using the random regression approach advocated here.

## Conclusion

Phenotypic plasticity is a central concept in evolutionary ecology (Schlichting 1986; Scheiner 1993; Schlichting and Pigliucci 1998). The seminal work of Weis and Gorman (1990) has clarified that quantification of natural selection on the level of the reaction norm is pivotal in understanding the evolution of plasticity. From this perspective, repeated observations on long-lived organisms under natural conditions in combination with the methodology we here presented constitute a powerful approach to quantify variation in the reaction-norm properties and linking them to variation in fitness.

## Supporting information

**Figure S1.** Plots of the Best Linear Unbiased Predictor (BLUP) values for the clutch size—laying date reaction-norm properties derived under the final model in Table 1 against relative fitness for 359 Ural owl females.
