# Supplementary figures and images for: Selection on plasticity of seasonal life-history traits using random regression mixed model analysis
Source: Ecol Evol. 2012 Apr;2(4):695–704. doi: 10.1002/ece3.60 (PMC3399192; doi:10.1002/ece3.60)

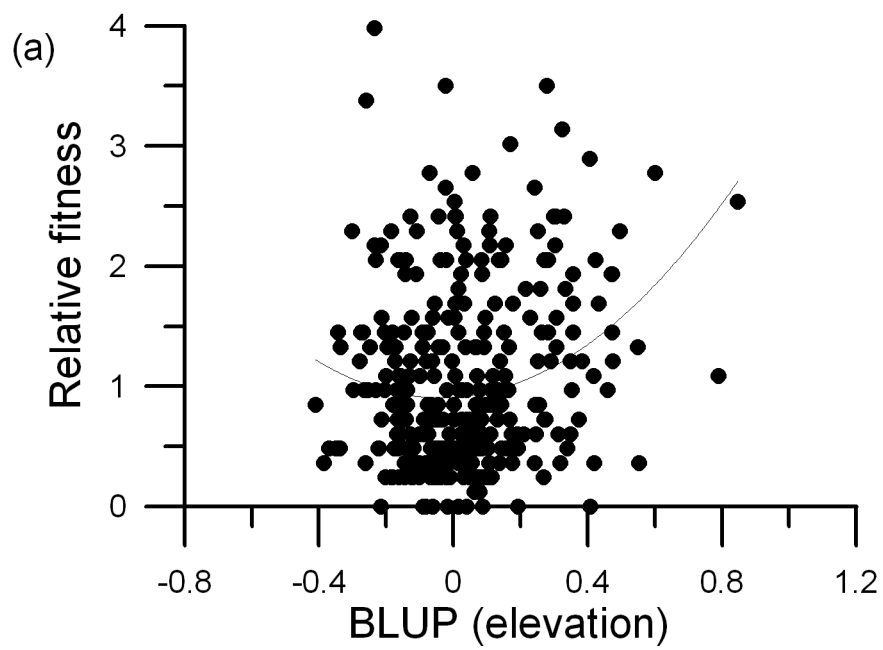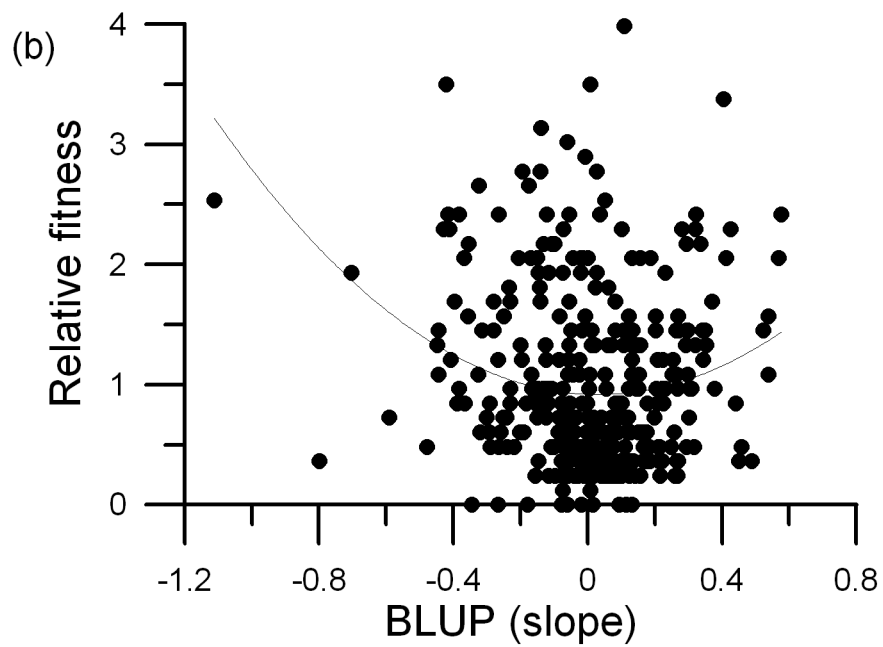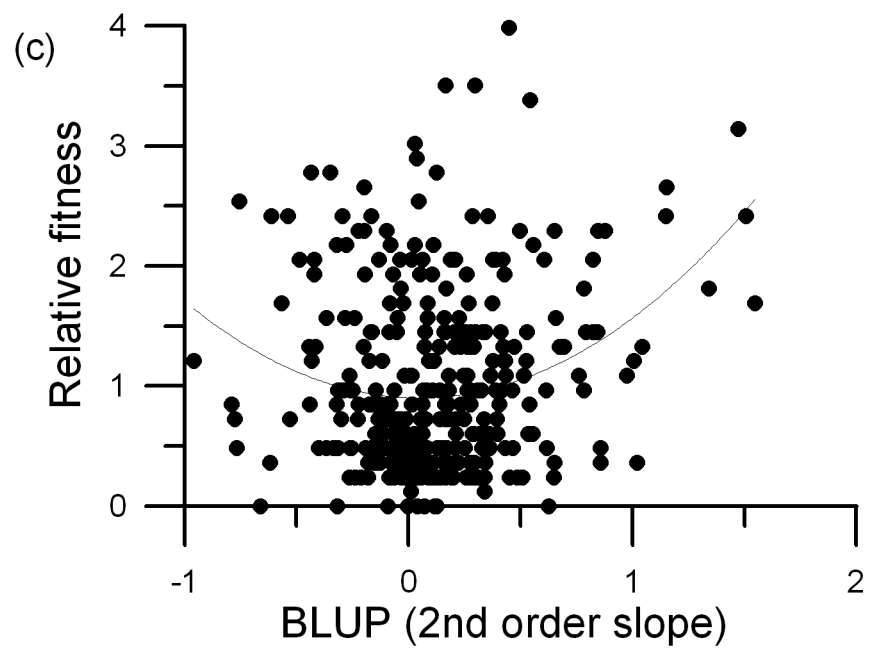

Supplement: Supplementary file 1 [file ece30002-0695-SD1.pdf]
